# RETRACTION: Bmi‐1 Plays a Critical Role in Protection From Renal Tubulointerstitial Injury by Maintaining Redox Balance

**DOI:** 10.1111/acel.70012

**Published:** 2025-02-12

## Abstract

**RETRACTION**: J. Jin, X. Lv, L. Chen, W. Zhang, J. Li, Q. Wang, R. Wang, X. Lu, and D. Miao, “Bmi‐1 Plays a Critical Role in Protection From Renal Tubulointerstitial Injury by Maintaining Redox Balance,” *Aging Cell* 13, no. 5 (2014): 797–809, https://doi.org/10.1111/acel.12236.

The above article, published online on 11 June 2014, in Wiley Online Library (wileyonlinelibrary.com), has been retracted by agreement between the journal Editor‐in‐Chief, Monty Montano; The Anatomical Society; and John Wiley & Sons Ltd. The retraction has been agreed upon following an investigation into concerns raised by a third party, which revealed image section duplications between this (Figure 3C, WT TNF‐α panel) and another article that was subsequently published by an overlapping group of authors, where the image depicts different experimental details. The investigation discovered further image duplications, showing overlapping fields of view between Figure S3G Cortex and Medulla KO panels and between Figure S4C and S4D Medulla KO TNF‐α and IL‐6 panels. The explanation and the partial raw data shared by the authors was deemed insufficient to fully address these concerns. Thus, the editors have lost confidence in the presented data and decided to retract the article. The authors disagree with the retraction.

**RETRACTION**: J. Jin, X. Lv, L. Chen, W. Zhang, J. Li, Q. Wang, R. Wang, X. Lu, and D. Miao, “Bmi‐1 Plays a Critical Role in Protection From Renal Tubulointerstitial Injury by Maintaining Redox Balance,” *Aging Cell* 13, no. 5 (2014): 797–809, https://doi.org/10.1111/acel.12236.

The above article, published online on 11 June 2014, in Wiley Online Library (wileyonlinelibrary.com), has been retracted by agreement between the journal Editor‐in‐Chief, Monty Montano; The Anatomical Society; and John Wiley & Sons Ltd. The retraction has been agreed upon following an investigation into concerns raised by a third party, which revealed image section duplications between this (Figure 3C, WT TNF‐α panel) and another article that was subsequently published by an overlapping group of authors, where the image depicts different experimental details. The investigation discovered further image duplications, showing overlapping fields of view between Figure S3G Cortex and Medulla KO panels and between Figure S4C and S4D Medulla KO TNF‐α and IL‐6 panels. The explanation and the partial raw data shared by the authors was deemed insufficient to fully address these concerns. Thus, the editors have lost confidence in the presented data and decided to retract the article. The authors disagree with the retraction.

